# Large next-generation sequencing gene panels in genetic heart disease: challenges in clinical practice

**DOI:** 10.1007/s12471-019-1251-4

**Published:** 2019-03-07

**Authors:** I. Christiaans, O. R. F. Mook, M. Alders, H. Bikker, R. H. Lekanne dit Deprez

**Affiliations:** 1 Department of Clinical Genetics, Amsterdam UMC, University of Amsterdam, Amsterdam, The Netherlands; 2 Department of Clinical Genetics, University Medical Centre Groningen, University of Groningen, Groningen, The Netherlands

**Keywords:** Next-generation sequencing, Variants of unknown significance, Cardiogenetics, Gene panel, Counselling

## Abstract

**Background:**

Next-generation sequencing gene panels are increasingly used for genetic diagnosis in inherited cardiac diseases. Besides pathogenic variants, multiple variants, variants of uncertain significance (VUS) and incidental findings can be detected. Such test results can be challenging for counselling and clinical decision making.

**Methods:**

We present patient cases to illustrate the challenges that can arise when unclear genetic test results are detected in cardiogenetic gene panels.

**Results:**

We identified three types of challenging gene panel results: 1) one or more VUS in combination with a pathogenic variant, 2) variants associated with another genetic heart disease, and 3) variants associated with a syndrome involving cardiac features.

**Conclusion:**

Large gene panels not only increase the detection rates of pathogenic variants but also of variants with uncertain pathogenicity, multiple variants and incidental findings. Gene panel results can be challenging for genetic counselling and require proper pre-test and post-test counselling. We advise evaluation of challenging cases by a multidisciplinary team.

## What’s new?

- Interpretation of genetic variants of unknown significance, multiple variants or incidental findings can be challenging.
- Doctors should be aware of these challenges and discuss them during pre-test counselling with their patient.
- It is important that challenging genetic test results are reviewed by a multidisciplinary team.

## Introduction

In inherited cardiac diseases next-generation sequencing (NGS) techniques with sequencing and analysis of multiple genes in a single experiment have become standard diagnostic care [1]. In the Netherlands, large gene panels analysing over 50 genes are offered for cardiomyopathies and for primary arrhythmia syndromes. Testing more genes using a gene panel or other NGS-based technique not only increases the detection rate of disease-causing variants, but also of variants of uncertain/unknown significance (VUS). VUS are variants whose implication with disease cannot be concluded based on current data. With time and further knowledge, VUS can then be classified as either benign without clinical significance or disease-causing.

With the more frequent use of NGS, genetic counsellors are increasingly being confronted with VUS. The goal of genetic testing is to provide the patient with more certainty about disease and prognosis, and, more importantly, in the case of genetic heart disease, to identify relatives at risk and enable possible treatments or preventive measures [2, 3]. The detection of a VUS clearly does not provide the genetic counsellor, the patient and his/her family with the desired certainty and the possibility of genetic cascade screening in relatives [4–8]. Interpretation and counselling can be even more challenging for clinicians without proper training in medical genetics, such as oncologists, cardiologists, or general practitioners, who may increasingly request NGS [9, 10]. Besides VUS, other unclear or unexpected findings can be detected using NGS-based techniques, such as multiple variants with different suspicions of pathogenicity and incidental findings (i. e. findings not related to the disease for which the test is requested). We use patient cases to illustrate different clinical challenges that derived from the cardiogenetic gene panel results. A separate article describes the yield of the cardiogenetic gene panels in terms of pathogenic variants and VUS.

## Methods

### Patients

Patients and family members were counselled and tested at the Cardiogenetics outpatient clinic of the Amsterdam UMC, University of Amsterdam, in a multidisciplinary setting including a detailed review of medical history, family history, cardiac evaluations, and counselling on possible outcomes of the gene panel for the patient and his/her relatives, including the possibility of detection of one or more VUS. We selected patients with an unclear test result of a cardiomyopathy or arrhythmia gene panel to illustrate the challenges associated with detection of variants with less clear pathogenicity. After an unclear genetic test result cases were discussed in a multidisciplinary team. All patients and family members gave informed consent for the anonymous use of their genetic data. The patients and families presented in the cases have been anonymised and partly changed to prevent identification.

### NGS-based cardiogenetic gene panel analyses and interpretation and classification of detected variants

Detailed information on the NGS-based gene panels is available in the accompanying article describing the yield in this issue and its online Supplementary File 1 [11]. Variants detected in a cardiogenetic gene panel in our laboratory are reported using Human Genome Variation Society nomenclature guidelines (http://www.hgvs.org/mutnomen) and classified into one of five categories (class 1: certainly not pathogenic, class 2: unlikely pathogenic, class 3: unknown pathogenicity (also called VUS), class 4: likely pathogenic; class 5: (certainly) pathogenic) using the classification criteria indicated in the online Supplementary File 1. Identified class 1 and 2 variants are not reported to the requesting physician.

## Results

Although both genetic counsellors and patients were aware of the possibility of VUS detection and associated uncertainty, they encountered challenges in counselling and clinical decision making in some patients. Here, we describe three types of challenges in counselling/clinical decision making derived from the gene panel results.

### VUS in a syndromic gene

A middle-aged female with idiopathic dilated cardiomyopathy (DCM) and a family history of premature sudden cardiac death was tested using the cardiomyopathy gene panel. We identified a class 3 variant in the *GLA* gene (c.1153A>G; p.(Thr385Ala), NM_000169.2). The variant changes a moderately conserved amino acid (PolyPhen HumDiv = 0.708 and HumVar = 0.122) and the chemical difference between Thr and Ala is small (Grantham distance: 58 (0–215)). At the time of the test result this variant had not been described in over 10,000 control alleles. The *GLA* gene on the X‑chromosome is one of the genes on the cardiomyopathy gene panel associated with a phenocopy of a cardiomyopathy. Pathogenic variants in the *GLA* gene cause Fabry disease, a lysosomal storage disorder characterised by neuropathic pain, angiokeratoma, chronic kidney disease, left ventricular hypertrophy (mimicking hypertrophic cardiomyopathy (HCM)), and cerebrovascular disease. Left ventricular hypertrophy can also be the only disease expression, especially in female carriers, and in carriers of specific *GLA* variants known to cause a cardiac-only phenotype. Patients suspected of HCM can have a *GLA* variant and clinical Fabry disease. Our patient, however, did not have HCM but DCM and her variant was of unknown pathogenicity. Possible explanations included a) that the *GLA* variant could be a new variant giving rise to a cardiac-only phenotype for Fabry disease; b) that our patient had Fabry disease with a DCM phenotype as an end-stage form of Fabry-related HCM/left ventricular hypertrophy [12]; or c) that the variant should be regarded as an incidental finding unrelated to the patient’s phenotype. Because enzyme replacement can be effective in Fabry disease, we had to decide whether to evaluate our patient for other features of Fabry disease, and also, how to counsel the family. We discussed the test result with our patient and asked for more details on possible Fabry symptoms in her and other relatives. She did not have any other symptoms of Fabry disease and neither did any of her relatives. She had two brothers who died suddenly in their forties, which could fit with an X‑linked inheritance, but they were said to have no symptoms specific to Fabry disease. We therefore decided Fabry disease was unlikely and considered the *GLA* variant an incidental finding. Because her DCM could still be hereditary, we advised regular cardiac evaluations for her first-degree relatives and the first-degree relatives of the sudden death victims in her family. Recently, this variant was reclassified to a class 2 variant (unlikely pathogenic). Reclassification was based on the frequency of this variant in controls with a similar ethnic background as our patient (53 times in 10,122 South Asian alleles) and a relatively high enzyme activity (45% of wild-type) in a functional study [13].

### Likely pathogenic variant in combination with a pathogenic variant

We performed DNA diagnostics using the cardiomyopathy gene panel in a middle-aged female with HCM without a family history of cardiomyopathy or sudden death. Her mother had developed atrial fibrillation at old age but showed no signs of HCM. We detected a pathogenic variant in the *MYBPC3* gene (c.2149-2del, NM_000256.3), but also a class 4 variant (likely pathogenic) in the *DSG2* gene (c.1143del; p.(Asn381Lysfs*2), NM_001943.3). The MYBPC3 gene is the most frequently mutated gene in HCM and we found a clear pathogenic variant. Variants in the *DSG2* gene are mainly associated with arrhythmogenic right ventricular cardiomyopathy (ARVC) and not with HCM. Rare nonsynonymous variants in desmosomal genes like DSG2 have been described in HCM cohorts in up to 20% of cases, but most of them are class 2 or 3 variants, often found in combination with a sarcomeric variant [14, 15]. Alfares et al. do not mention class 4 or 5 variants in desmosomal genes in their large cohort of HCM patients [16]. Still, a class 4 variant in this gene should be taken seriously. This specific DSG2 variant has not been described previously in literature and was not observed in about 120,000 alleles from the Exome Aggregation Consortium (ExAC) database. Although this variant results in a truncated protein, this does not imply it is certainly pathogenic. Truncating variants in DSG2 are also observed in controls, but about two times more frequent in ARVC patients (https://cardiodb.org/ACGV/).

This test result could mean that a) the *DSG2* variant contributes to the HCM phenotype of our patient; b) the patient now also has an increased risk of ARVC; or c) the *DSG2* variant is benign and does not cause disease. We had to decide whether to offer relatives predictive genetic testing for the *MYBPC3* variant alone or for the *DSG2* variant as well. The risk of cardiac disease (ARVC or HCM) for relatives only carrying the *DSG2* variant remained unknown. We therefore chose a practical approach and offered the family of this patient predictive genetic testing for both variants. Carriers of one or two of the variants were advised to have regular cardiac evaluations.

### Variant in a gene associated with other disease

A young infant showed signs of cardiomyopathy (a combination of restrictive and dilated cardiomyopathy) and was tested using the cardiomyopathy gene panel. We identified a clear pathogenic variant in the *SCN5A* gene (c.5228G>A; p.(Gly1743Glu), NM_001099404.1). Although variants in *SCN5A* are associated with dilated cardiomyopathy, this specific variant was known to be associated with Brugada syndrome, not with DCM. The nucleotide change c.5228G>A predicts the amino acid substitution p.(Gly1743Glu) in the SCN5A protein. The variant changes a highly conserved amino acid (PolyPhen HumDiv = 1 and HumVar = 1) and the chemical difference between Gly and Glu is moderate (Grantham distance: 98 (0–215)). The variant has been described in Brugada patients [17, 18]. Functional studies in a heterologous expression system showed a reduced sodium current compared with wildtype [19]. In addition, the variant was not observed in about 121,000 alleles from the ExAC database.

The detection of this *SCN5A* variant could mean: a) that this child now (also) has Brugada syndrome: or b) that this *SCN5A* variant can also cause DCM, possibly as an end-stage result of tachyarrhythmia. The majority of Brugada syndrome patients are diagnosed in their forties, but ECG abnormalities can already be seen in childhood. Some variants have been associated with sudden infant death syndrome (SIDS). This specific variant has not been described to result in an atypical form of Brugada syndrome. Of note, our DNA laboratory found this variant in several Brugada patients, but also in one SIDS case. This child did not show any arrhythmia or Brugada signs on the ECG and was too young to perform a provocation test with ajmaline or flecainide. DCM as an end-stage result of a severe Brugada phenotype was therefore unlikely. Clinically, the child does not have Brugada syndrome yet, but is probably at risk of developing it. We decided to first counsel the parents and offer them cardiac evaluation and DNA testing to possibly support our thoughts. The mother of the child showed signs of Brugada syndrome on her ECG and DNA testing showed that she was a carrier of the *SCN5A* variant. We then concluded that the *SCN5A* variant was indeed related to Brugada syndrome in this family and offered predictive genetic testing to the first-degree relatives of the mother. All of them were tested and several were found to be carrier of the *SCN5A* variant. We assume the DCM of the infant is not explained by the *SCN5A* variant and because we cannot exclude a genetic cause, we advised cardiac evaluations to the child’s parents and siblings.

## Discussion

Our gene panels have an overall detection rate (class 3, 4, and 5 variants) of around 50%. Most of the detected variants are variants of uncertain significance. The high frequency of these VUS (in about 30–40% of cardiogenetic gene panel test results) necessitates pre-test counselling on VUS of tested patients. In our centre, all patients receive such pre-test counselling by a genetic counsellor specialised in cardiogenetics and with knowledge of (counselling) VUS. Still, we encountered unexpected results giving rise to challenges in counselling and clinical decision making. We identified three types of challenging gene panel results: 1) one or more VUS in combination with a pathogenic variant; 2) variants associated with another genetic heart disease; and 3) variants associated with a syndrome involving cardiac features. Therefore, pre-test counselling—even in disease-specific gene panels—should not only include information on disease-related VUS, but also on incidental findings (i. e. not directly related with the disease tested for). In our experience, and literature supports this, genetic test results, especially VUS, are not only difficult to understand for a patient, but are also often misinterpreted by non-genetic physicians [20–22]. We therefore also recommend pre-test and post-test counselling to be performed by a physician/counsellor with sufficient knowledge on VUS and variant classification. Knowledge, understanding, and education on VUS and other possible genetic test results are certainly areas for further study.

As illustrated in the cases the unclear test results are discussed in a multidisciplinary team to review the case from different angles. This team consists of clinical geneticists, cardiologists, clinical molecular geneticists, and researchers all specialised in cardiogenetics. Follow-up of patients and family members may be necessary to evaluate decisions that have been made. In our opinion it is important that physicians requesting cardiogenetic NGS-based tests are involved in or are in contact with such a multidisciplinary team.

## Conclusions

The use of larger NGS-based gene panels does not only increase the detection rates of pathogenic variants but also of multiple variants, variants of uncertain significance and incidental findings. Such test results, especially VUS and incidental findings, can be challenging for genetic counselling and call for proper pre-test and post-test counselling. We advise the evaluation of challenging cases by a multidisciplinary team.
